# Asn 362 in gp120 contributes to enhanced fusogenicity by CCR5-restricted HIV-1 envelope glycoprotein variants from patients with AIDS

**DOI:** 10.1186/1742-4690-4-89

**Published:** 2007-12-12

**Authors:** Jasminka Sterjovski, Melissa J Churchill, Anne Ellett, Lachlan R Gray, Michael J Roche, Rebecca L Dunfee, Damian FJ Purcell, Nitin Saksena, Bin Wang, Secondo Sonza, Steven L Wesselingh, Ingrid Karlsson, Eva-Maria Fenyo, Dana Gabuzda, Anthony L Cunningham, Paul R Gorry

**Affiliations:** 1 Macfarlane Burnet Institute for Medical Research & Public Health, Melbourne, Victoria, Australia; 2 Department of Medicine, Monash University, Melbourne, Victoria, Australia; 3 Department of Microbiology, Monash University, Melbourne, Victoria, Australia; 4 Department of Microbiology and Immunology, University of Melbourne, Melbourne, Victoria, Australia; 5 Dana-Farber Cancer Institute, Boston, MA, USA; 6 Westmead Millennium Institute, Westmead, New South Wales, Australia; 7 Lund University, Lund, Sweden; 8 Department of Neurology, Harvard Medical School, Boston, MA, USA

## Abstract

**Background:**

CCR5-restricted (R5) human immunodeficiency virus type 1 (HIV-1) variants cause CD4+ T-cell loss in the majority of individuals who progress to AIDS, but mechanisms underlying the pathogenicity of R5 strains are poorly understood. To better understand envelope glycoprotein (Env) determinants contributing to pathogenicity of R5 viruses, we characterized 37 full-length R5 Envs from cross-sectional and longitudinal R5 viruses isolated from blood of patients with asymptomatic infection or AIDS, referred to as pre-AIDS (PA) and AIDS (A) R5 Envs, respectively.

**Results:**

Compared to PA-R5 Envs, A-R5 Envs had enhanced fusogenicity in quantitative cell-cell fusion assays, and reduced sensitivity to inhibition by the fusion inhibitor T-20. Sequence analysis identified the presence of Asn 362 (N362), a potential N-linked glycosylation site immediately N-terminal to CD4-binding site (CD4bs) residues in the C3 region of gp120, more frequently in A-R5 Envs than PA-R5 Envs. N362 was associated with enhanced fusogenicity, faster entry kinetics, and increased sensitivity of Env-pseudotyped reporter viruses to neutralization by the CD4bs-directed Env mAb IgG1b12. Mutagenesis studies showed N362 contributes to enhanced fusogenicity of most A-R5 Envs. Molecular models indicate N362 is located adjacent to the CD4 binding loop of gp120, and suggest N362 may enhance fusogenicity by promoting greater exposure of the CD4bs and/or stabilizing the CD4-bound Env structure.

**Conclusion:**

Enhanced fusogenicity is a phenotype of the A-R5 Envs studied, which was associated with the presence of N362, enhanced HIV-1 entry kinetics and increased CD4bs exposure in gp120. N362 contributes to fusogenicity of R5 Envs in a strain dependent manner. Our studies suggest enhanced fusogenicity of A-R5 Envs may contribute to CD4+ T-cell loss in subjects who progress to AIDS whilst harbouring R5 HIV-1 variants. N362 may contribute to this effect in some individuals.

## Background

The gp120 and gp41 envelope glycoprotein (Env) complexes of human immunodeficiency virus type 1 (HIV-1) mediate viral entry into cells (reviewed in [1-3]). The gp120 subunits bind to CD4 which induces conformational changes that lead to exposure of a binding site for a cellular coreceptor, either CCR5 or CXCR4. Coreceptor binding induces further conformational changes in gp41 that lead to fusion between the viral and cellular membranes and entry of the HIV-1 core into cells.

The coreceptor specificity of Env influences HIV-1 pathogenesis. Progression of HIV-1 infection from early, asymptomatic stages of disease to acquired immunodeficiency syndrome (AIDS) is associated with a switch in viral coreceptor specificity from CCR5-using (R5) viral strains to those able to use CXCR4 (X4) or both coreceptors (R5X4) in 40–50% of infected adults [4-8] (reviewed in [9]). However, X4 or R5X4 variants are absent in 50–60% of HIV-1 infected individuals who progress to AIDS [10-14] (reviewed in [15]). Therefore, the persistence of an exclusive R5 viral population *in vivo *is sufficient to cause immunodeficiency in the majority of HIV-1 infected individuals who progress to AIDS.

In addition to dictating HIV-1 coreceptor specificity, the Env glycoproteins cause significant cytotoxicity both *in vitro *and *in vivo*. Env mediates most of the acute cytopathic effects of HIV-1 infection in cultured cells [16], and membrane fusion appears to be an important factor contributing to HIV-1 cytopathicity *in vitro *[17]. Passage of chimeric simian-HIV (SHIV) strains in macaques demonstrated enhancement of pathogenicity that was associated with mutations in Env [18-23]. These Env mutations often resulted in increased Env-mediated membrane fusing capacity [20,23-26], suggesting that fusogenicity contributes to viral pathogenicity in this animal model. The cytopathic effects of Env-mediated HIV-1 fusogenicity are also evident in humans. For example, the presence of multinucleated giant cells (MNGC) in brain, formed by Env-mediated fusion between infected and uninfected macrophage lineage cells, is characteristic of HIV-1 encephalitis (HIVE) and a neuropathological hallmark of HIV-associated dementia (reviewed in [27]). Thus, Env-mediated fusogenicity appears to be an important factor contributing to HIV-1 pathogenesis.

Whilst much effort has been directed towards understanding the molecular basis of pathogenicity of late-emerging X4 and R5X4 viruses [28-30] (reviewed in [9]), the molecular mechanisms underlying the pathogenicity of R5 HIV-1 strains are poorly understood [15]. R5 viruses are intrinsically cytopathic, but exert pathogenic effects that are distinct from those of X4 or R5X4 viruses [31-33]. R5 HIV-1 strains isolated from patients with AIDS (hereafter referred to as AIDS R5 (A-R5) viruses) have enhanced macrophage (M)-tropism [34-36] and cause increased levels of CD4+ T-cell death [37] compared with R5 HIV-1 strains isolated from asymptomatic individuals (hereafter referred to as pre-AIDS R5 (PA-R5) viruses). A-R5 viruses were shown to have increased *in vivo *cytopathicity in HIV-1-infected SCID-hu mice compared with PA-R5 viruses in one study [38], although different conclusions were reached by other *in vivo *and *ex vivo *studies [39,40]. A-R5 viruses have decreased sensitivity to inhibition by the β-chemokine RANTES (Regulated on Activation, Normally T-cell-expressed and -secreted) compared with PA-R5 viruses [10,13,14]. Recent evidence suggests that decreased RANTES sensitivity is attributed to an increased flexibility of the R5 Env that alters the mode and efficiency of CCR5 usage [13]. In addition, A-R5 viruses have decreased sensitivity to inhibition by the HIV-1 fusion inhibitor T-20 and by the CCR5 antagonist TAK-779 compared with PA-R5 viruses [36,41], but have increased sensitivity to neutralization by the CD4 binding site (CD4bs) directed Env monoclonal antibody (mAb) IgG1b12 [36]. Together, these findings provide evidence that A-R5 viruses have intrinsic properties distinguishing them from PA-R5 viruses which may enhance their cytopathic effects, and that these properties are likely to be linked to Env conformations that enhance CD4 and/or CCR5 interactions.

Genetic determinants of the Env underlying these A-R5 HIV-1 phenotypes, which may contribute to HIV-1 pathogenesis in subjects who persistently harbor R5 HIV-1 variants to late stages of HIV-1 infection are unknown. To better understand Env determinants contributing to pathogenicity of R5 viruses, we characterized R5 Envs generated from cross-sectional and longitudinal panels of PA-R5 and A-R5 viruses. Our results show that enhanced fusogenicity is a phenotype of A-R5 Envs. We identified the presence of Asn 362 (N362), a potential N-linked glycosylation site immediately N-terminal to CD4bs residues in the C3 region of gp120, more frequently in A-R5 Envs than PA-R5 Envs. N362 was associated with enhanced fusogenicity, reduced sensitivity to the inhibitory effects of T-20, faster entry kinetics, and increased sensitivity of Env-pseudotyped reporter viruses to neutralization by the CD4bs-directed Env mAb IgG1b12. Mutagenesis studies showed that N362 contributes to fusogenicity of R5 Envs in a strain dependent manner. Structural models indicate that N362 is located adjacent to the CD4 binding loop of gp120, and suggest N362 may contribute to enhanced fusogenicity by promoting greater exposure of the CD4bs and/or stabilizing the CD4-bound Env structure. This prediction is consistent with the increased sensitivity of A-R5 Envs with N362 to neutralization by IgG1b12. Enhanced fusogenicity of A-R5 Envs may contribute, at least in part, to CD4+ T-cell loss in subjects who progress to AIDS whilst harbouring R5 HIV-1 variants.

## Results

### Primary PA-R5 and A-R5 HIV-1 isolates

We characterized HIV-1 Envs cloned from a series of well characterized primary PA-R5 and A-R5 viruses. These included a cross sectional panel of four PA-R5 viruses (NB23, NB24, NB25 and NB27) and four A-R5 HIV-1 viruses (NB2, NB6, NB7 and NB8) [34,36], as well as PA-R5 and A-R5 viruses isolated sequentially from one subject (IK1) [13,42] (Table 1). All viruses are of R5 phenotype, but compared to the PA-R5 viruses the A-R5 viruses have enhanced M-tropism, reduced CD4- and CCR5-dependence, reduced sensitivity to inhibition by HIV-1 entry inhibitors and RANTES, and increased sensitivity to neutralization by the Env mAb IgG1b12 [13,34,36,42]. Thus, the A-R5 HIV-1 isolates have unique biological properties distinguishing them from the PA-R5 isolates that serve to enhance Env-receptor interactions, and most likely map to the *env *gene. However, it is important to note that the PA-R5 virus from subject IK1 was isolated just prior to the onset of CD4+ T-cell loss and progression toward AIDS [13], whereas the PA-R5 viruses from the cross sectional panel were isolated at earlier stages of HIV-1 infection, including one virus isolated from an acute seroconverter [34]. Therefore, although all the A-R5 viruses were isolated from patients with AIDS, there is considerable heterogeneity among the PA-R5 viruses with respect to the stage of asymptomatic HIV-1 infection from which they were isolated.

**Table 1:** Characteristics of primary R5 viruses and Env clones

**Virus**^a^	**Description**^b^	**Env clone**^c^	**Coreceptor usage**^d^	
			**CCR5**	**CXCR4**
**Cross-sectional viruses**				-
NB23	PA-R5	NB23-C1	+++	-
		NB23-C2	+++	-
		NB23-C3	+++	-
NB24	PA-R5	NB24-C1	++	-
		NB24-C2	+	-
		NB24-C3	+	-
		NB24-C4	+	-
NB25	PA-R5	NB25-C1	+	-
		NB25-C2	+	-
		NB25-C3	+++	-
NB27	PA-R5	NB27-C1	+	-
		NB27-C2	++	-
		NB27-C3	+++	-
NB2	A-R5	NB2-C1	+	-
		NB2-C2	+	-
		NB2-C3	++	-
		NB2-C4	++	-
NB6	A-R5	NB6-C1	+++	-
		NB6-C2	+++	-
		NB6-C3	+++	-
		NB6-C4	+++	-
NB7	A-R5	NB7-C1	++	-
		NB7-C2	++	-
		NB7-C3	++	-
		NB7-C4	++	-
NB8	A-R5	NB8-C1	+++	-
		NB8-C2	+++	-
		NB8-C3	+++	-
		NB8-C4	+++	-

**Longitudinal viruses**				
IK1-PA	PA-R5	IK1-PA-C1	++	-
		IK1-PA-C2	+	-
		IK1-PA-C3	++	-
		IK1-PA-C4	+	-
IK1-A	A-R5	IK1-A-C1	++	-
		IK1-A-C2	+++	-
		IK1-A-C3	++	-
		IK1-A-C4	++	-

**Controls**				
		ΔKS Env	-	-
		HXB2 Env	-	+++
		ADA Env	+++	-
		89.6 Env	+++	+++

^a^The phenotypes of the primary R5 HIV-1 isolates, and clinical characteristics of the subjects from whom they were isolated have been described in detail previously [11, 13, 34, 36, 42].

^b^PA-R5, pre-AIDS R5 HIV-1 isolate; A-R5, AIDS R5 HIV-1 isolate.

^c^Functional Env clones were identified by infection of JC53 cells or Cf2-CD4/CCR5/CXCR4 cells with Env-pseudotyped GFP reporter viruses and by fusion assays, as described in the Methods (data not shown).

^d^Coreceptor usage of functional Envs was determined by infection of Cf2-CD4/CCR5 and Cf2-CD4/CXCR4 cell lines with Env-pseudotyped GFP-reporter viruses, as described in the Methods. GFP positive cells were counted manually by fluorescence microscopy and scored as – (no GFP positive cells), +/- (1 to 5% GFP positive cells), + (5 to 10% GFP positive cells), ++ (10 to 30% GFP positive cells), or +++ (>30% GFP positive cells).

### Biological activities of HIV-1 Env clones

**Figure 1:**
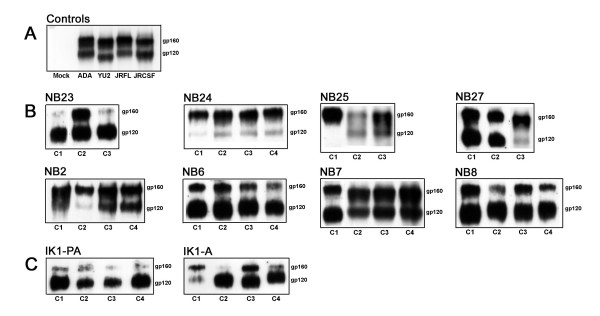
**Expression of functional Env clones**. 293T cells were cotransfected with 8 μg of pSVIII-Env plasmid expressing control R5 Envs (A) or pSVIII-Env plasmid expressing functional Envs cloned from the cross sectional (PA-R5 viruses NB23, NB24, NB25, NB27 and A-R5 viruses NB2, NB6, NB7, NB8) (B) or longitudinal (PA- and A-R5 viruses from subject IK1) (C) primary R5 HIV-1 isolates and 2 μg pSVL-Tat, as described in the Methods. Env expression at 72 h post-transfection was measured by Western blot analysis of cell lysates using rabbit anti-gp120 polyclonal antisera. Positions of gp160 and gp120 are shown on the right. C1, C2, C3 and C4 refer to independent Envs cloned from each virus.

To identify viral determinants which underlie the unique biological properties of A-R5 HIV-1 viruses and may contribute to the pathogenesis of R5 HIV-1 variants, the *env *gene was cloned into the pSVIII-HXB2 Env expression vector using *Kpn*I and *Bam*HI restriction sites. Three to four independent and functional Envs cloned from each virus were identified by single round entry assays in JC53 or Cf2-CD4/CCR5/CXCR4 cells using Env-pseudotyped GFP reporter virus, and by fusion assays (Table 1, and data not shown). Western blot analysis of Env expression in transfected 293T cells showed distinct gp160 and gp120 proteins in 36/37 primary Envs, similar to the control R5 ADA, YU2, JRFL and JRCSF Envs (Fig. 1). To determine the coreceptor specificity of the cloned Envs, Env-pseudotyped GFP reporter viruses were used in single round entry assays with Cf2th cell lines stably expressing CD4/CCR5 or CD4/CXCR4 (Table 1). The X4 HXB2, R5 ADA, and R5X4 89.6 Envs were used as positive controls. A non functional Env, ΔKS Env, was used as a negative control to determine background levels of GFP expression. As expected, HXB2 Env used CXCR4, ADA Env used CCR5, and 89.6 Env used both CXCR4 and CCR5 for HIV-1 entry. All 37 primary Envs used CCR5 for HIV-1 entry, similar to the coreceptor specificity of the primary isolates from which they were cloned. Thus, we established and validated a bank of functional PA-R5 and A-R5 Envs cloned from well characterized primary R5 HIV-1 isolates.

### A-R5 Envs have enhanced fusogenicity compared to PA-R5 Envs

**Figure 2:**
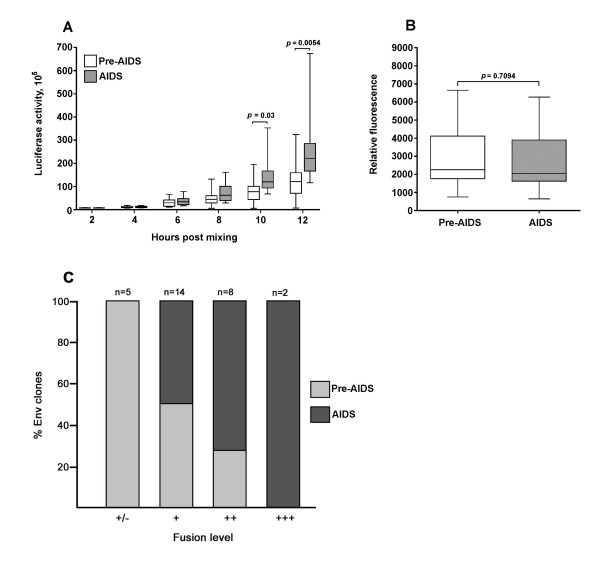
**Fusogenicity of PA-R5 and A-R5 Envs cloned from the cross-sectional panel of primary R5 HIV-1 isolates**. Fusion assays were performed using 293T effector cells expressing PA-R5 and A-R5 Envs shown in Fig. 1B and Cf2-Luc target cells expressing CD4 and CCR5, as described in the Methods. Cells were harvested at 2, 4, 6, 8, 10 and 12 h post-mixing and assayed for luciferase activity (A). 293T effector cells were stained for cell surface Env expression using pooled AIDS serum and analysed by flow cytometry, as described in the Methods (B). The data were stratified by different maximal levels of fusion scored as +/-, +, ++, and +++, which correspond to <10-fold (very low), 10- to 20-fold (low), 20- to 40-fold (moderate), and >40-fold (high) increases in luciferase activity above background levels, respectively (C). Box plots were constructed from mean values of duplicate experiments with each Env using Prism version 4.0c (GraphPad Software, San Diego, CA.). Boxes represent upper and lower quartiles and median scores, and whiskers represent minimum and maximum values. The data shown are representative of 3 independent experiments. P values were calculated using a nonparametric Mann-Whitney *U *test, and values <0.05 were considered statistically significant.

Alterations in Env that augment fusogenicity contribute to the pathogenesis of SHIV infection [20,23-26]. In addition, HIV-1 fusogenicity is evident as MNGCs in tissues such as brain, which frequently harbors highly fusogenic R5 HIV-1 strains that share a number of phenotypic characteristics with blood-derived A-R5 viruses [43-46]. We used a quantitative cell-cell fusion assay to determine whether A-R5 Envs are more fusogenic than PA-R5 Envs. In this assay, cells were sampled at 2-hourly intervals until maximal fusion levels were reached at 12 hours post-mixing of Env-expressing effector cells and CD4/CCR5-expressing target cells. A-R5 Envs from the cross-sectional panel caused greater levels of cell-cell fusion than the PA-R5 Envs, which was particularly evident at 10 and 12 h post-mixing (Fig. 2A). The differences in fusogenicity between PA- and A-R5 Envs were not due to differences in cell surface Env expression levels on effector cells (Fig. 2B). When maximal fusion levels attained by PA- and A-R5 Envs were stratified across very low (+/-), low (+), moderate (++) or high (+++) maximal levels, the majority of A-R5 Envs had either moderate or high maximal fusion levels, whereas the majority of PA-R5 Envs had either very low or low maximal fusion levels (Fig. 2C). Thus, the A-R5 Envs from the cross-sectional panel are more fusogenic than the PA-R5 Envs.

**Figure 3:**
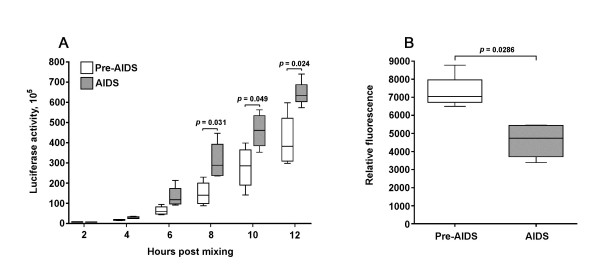
**Fusogenicity of PA-R5 and A-R5 Envs cloned from longitudinal primary R5 HIV-1 isolates**. Fusion assays were performed using 293T effector cells expressing PA-R5 and A-R5 Envs cloned from longitudinal viruses isolated from subject IK1 shown in Fig. 1C, and Cf2-Luc target cells expressing CD4 and CCR5 as described in the Methods. Cells were harvested at 2, 4, 6, 8, 10 and 12 h post-mixing and assayed for luciferase activity (A). 293T effector cells expressing Envs were stained for cell surface Env expression using pooled AIDS serum and analysed by flow cytometry, as described in the Methods (B). Box plots were constructed from mean values of duplicate experiments with each Env using Prism version 4.0c (GraphPad Software). Boxes represent upper and lower quartiles and median scores, and whiskers represent minimum and maximum values. The data shown are representative of 3 independent experiments. P values were calculated using a nonparametric Mann-Whitney *U *test, and values < 0.05 were considered statistically significant.

We next tested whether A-R5 Envs from IK1 are more fusogenic than PA-R5 Envs cloned from this subject. A-R5 Envs from IK1 caused greater levels of cell-cell fusion than matched PA-R5 Envs, which was evident at 8, 10 and 12 h post-mixing (Fig. 3A). The differences in fusogenicity between the PA-R5 and A-R5 Envs were not due to differences in cell surface Env expression levels on effector cells (Fig. 3B). In fact, in these experiments PA-R5 Envs were expressed to greater levels on effector cells than A-R5 Envs. The extent of cell-cell fusion is directly related to the level of Env expression on effector cells (J. Sterjovski and P.R. Gorry, unpublished data). Thus, in subject IK1, A-R5 Envs are more fusogenic than PA-R5 Envs, and the differences shown in Figure 3A are likely to be conservative.

### A-R5 Envs are less sensitive to inhibition by the HIV-1 fusion inhibitor T-20 than PA-R5 Envs

**Figure 4:**
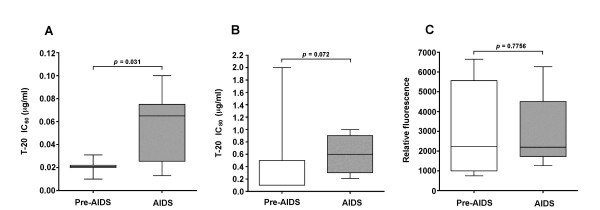
**Sensitivity of PA-R5 and A-R5 Envs to inhibition by T-20**. Fusion assays were performed using 293T effector cells expressing PA-R5 and A-R5 Envs from the cross sectional panel of primary R5 HIV-1 isolates shown in Fig. 1A, and Cf2-Luc target cells expressing CD4 and CCR5 in the presence of 10-fold increasing concentrations of T-20 ranging from 0.001 to 10 μg per ml, as described in the Methods. IC_50 _(A) and IC_80 _(B) values were calculated by least squares analysis of inhibition curves. IC_80 _values were calculated instead of IC_90 _values, because 90% inhibition of fusion was not reached when these concentrations of T-20 were tested against some of the A-R5 Envs (data not shown). 293T effector cells were stained for cell surface Env expression using pooled AIDS serum and analysed by flow cytometry, as described in the Methods (C). Box plots were constructed from mean values of duplicate experiments with each Env using Prism version 4.0c (GraphPad Software). Boxes represent upper and lower quartiles and median scores, and whiskers represent minimum and maximum values. The data shown are representative of 3 independent experiments. P values were calculated using a nonparametric Mann-Whitney *U *test, and values < 0.05 were considered statistically significant.

Enhanced fusogenicity of A-R5 Envs suggests that these Envs may be less sensitive to the antiviral effects of the HIV-1 fusion inhibitor T-20 than PA-R5 Envs. Therefore, we next determined the sensitivity of A-R5 and PA-R5 Envs from the cross sectional viruses to inhibition by T-20. Quantitative cell-cell fusion assays were carried out in the presence of 10-fold increasing concentrations of T-20 ranging from 0.001 to 10 μg per ml, and IC_50 _and IC_80 _values calculated by regression analysis of inhibition curves. The results demonstrate a significant increase in the IC_50 _(Fig. 4A) and a non-significant trend toward an increase in the IC_80 _(Fig. 4B) for T-20 against A-R5 Envs compared to PA-R5 Envs. These differences were not due to differences in cell surface Env expression levels on effector cells (Fig. 4C). Thus, highly fusogenic A-R5 Envs appear to be less sensitive to the inhibitory effects of T-20 in cell-cell fusion assays compared to less fusogenic PA-R5 Envs.

### N362 adjacent to CD4bs residues in gp120 is conserved in A-R5 but not PA-R5 Envs

To identify amino acid variants associated with A-R5 Envs that may contribute to enhanced fusogenicity, the gp120 region of the 37 Envs was sequenced and analyzed. Phylogenetic analysis of Envs demonstrated tight clustering of nucleotide sequences according to virus isolate (data not shown). Multiple sequence alignments verified that all sequences were unique (data not shown). Together, these data demonstrate that the Envs are independent clones and not the result of sequence resampling.

A-R5 and PA-R5 Envs could not be segregated based on the total number of potential N-linked glycosylation sites (PNGS) in gp120 (range, 16 to 24 PNGS; median 19), length of the V1V2 variable loops (range, 68 to 83 amino acids; median 72), net charge of the V1V2 (range, -3 to +4; median +1) or V3 (range, +3 to +8, median +5) amino acid sequence, or number of PNGS in the V4 (range, 3 to 6 PNGS, median 4) or V5 (range 0 to 2 PNGS, median 1) sequence (data not shown), which are parameters shown previously to affect the biological activity of HIV-1 Envs [47-55]. Net charge of the V3 variable loop region did not predict coreceptor usage, consistent with results of previous studies [44,46,56,57].

**Figure 5:**
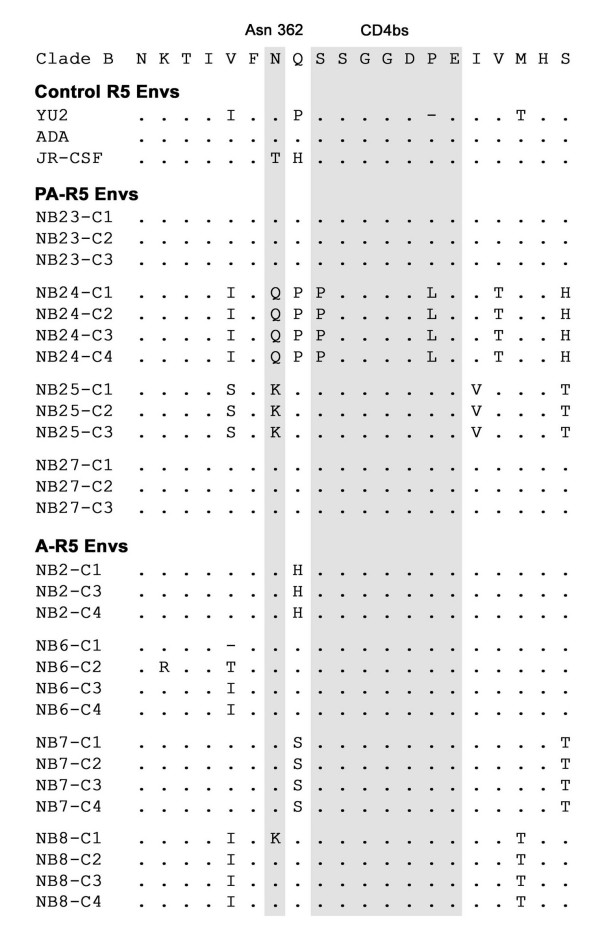
**Amino acid sequences spanning the CD4bs in the C3 region of gp120**. Amino acid alignments of the C3 region of PA-R5 and A-R5 Envs cloned from the cross sectional panel of primary HIV-1 isolates are compared to those from the highly fusogenic YU2 and ADA R5 Envs, the poorly fusogenic JR-CSF R5 Env, and the clade B consensus sequence. Dots indicate residues identical to the clade B consensus sequence, and dashes indicate gaps. Residues forming the CD4bs and the amino acid present at position 362 (numbered relative to the HXB2 reference sequence) are highlighted.

Signature pattern analysis of A-R5 and PA-R5 Envs from the cross-sectional viruses identified an amino acid variant, N362, that was present more frequently in A-R5 Envs (14/15 Envs; 93%) than PA-R5 Envs (6/13 Envs; 46%) (Fig. 5). No additional Env changes that could potentially distinguish A-R5 Envs from PA-R5 Envs were identified. Consistent with these results, database analysis of published Env sequences where sufficient clinical information was present to confidently assign Envs as A-R5 or PA-R5 [34,38,58-65] demonstrated N362 is significantly more frequent in A-R5 Envs (74%; n = 142) compared with PA-R5 Envs (49%; n = 77) (p = 0.0004, Fisher's exact test). N362 is located in the C3 Env region immediately N-terminal to residues in the CD4bs [50], suggesting that N362 could potentially influence Env-CD4 binding. N362 is also present in ADA and YU2 R5 Envs, which are highly fusogenic, similar to the majority of A-R5 Envs (data not shown). In contrast, threonine is present at this position in JR-CSF Env, which is poorly fusogenic, similar to the majority of PA-R5 Envs (data not shown). Thus, the presence of N362 is associated with A-R5 Envs from the cross-sectional panel and other published Env sequences, and may contribute to enhanced fusogenicity. However, since N362 is present in a relatively high proportion of PA-R5 Envs from the cross sectional viruses (6/13) and other published studies (49%), any effect N362 may have on the biological activity of A-R5 Envs is likely to be strain-specific and/or context dependent.

### N362 is associated with enhanced fusogenicity and reduced sensitivity to inhibition by T-20

**Figure 6:**
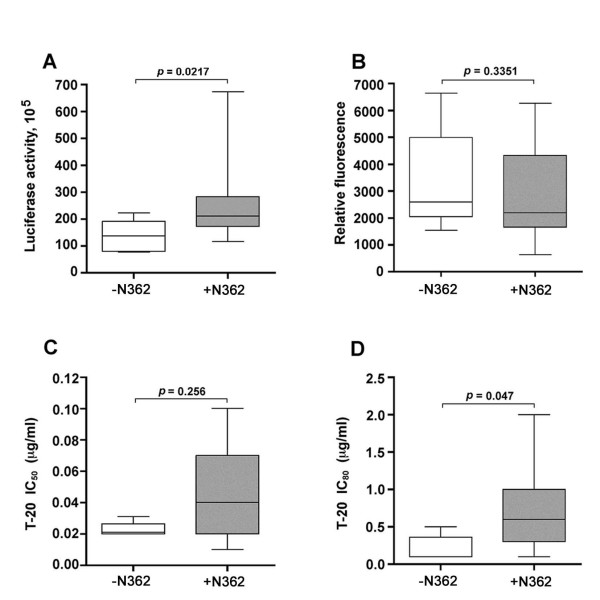
**N362 is associated with enhanced fusogenicity and reduced sensitivity to T-20**. For each of the Envs cloned from the cross sectional panel of primary R5 HIV-1 viruses, the maximal levels of fusion (determined at 12 h post-fusion) (A), cell surface Env expression on 293T effector cells (B), and IC_50 _(C) and IC_80 _(D) values for sensitivity to inhibition by T-20, were stratified based on the presence or absence of N362. Box plots were constructed from mean values of duplicate experiments with each Env using Prism version 4.0c (GraphPad Software). Boxes represent upper and lower quartiles and median scores, and whiskers represent minimum and maximum values. The data shown are representative of 3 independent experiments.

The preceding studies showed a predominance of A-R5 Envs containing N362, but Envs from two PA-R5 viruses, NB23 and NB27, also contained N362. Furthermore, although the fusogenicity of A-R5 Envs from the cross-sectional panel was significantly greater than that of the PA-R5 Envs, there was still considerable overlap. To better understand the relationship between the presence of N362, fusogenicity, and sensitivity to T-20, these data were stratified based on the presence or absence of N362 (Fig. 6). R5 Envs containing N362 had significantly greater maximal levels of cell-cell fusion than R5 Envs lacking N362 (Fig. 6A). The differences in cell-cell fusion between Envs containing or lacking N362 were not due to differences in cell surface Env expression levels on effector cells (Fig. 6B). Envs containing N362 had a significantly higher IC_80 _and a trend toward a higher IC_50 _for T-20 than Envs lacking N362 (Fig. 6C,D). Together, these data demonstrate an association between the presence of N362 in R5 Envs from the cross sectional panel and enhanced fusogenicity, and an additional association between the presence of N362 and sensitivity to the inhibitory effects of T-20 in cell-cell fusion assays.

### Molecular modeling of N362

**Figure 7:**
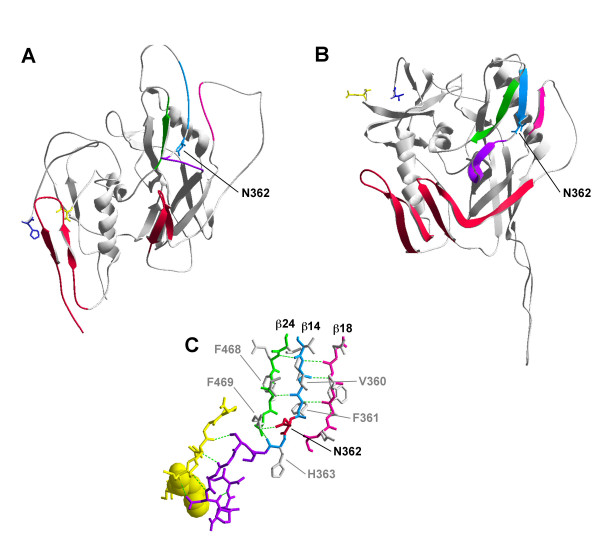
**Structural modelling of N362**. Structures of the unliganded SIV gp120 (A) and CD4-bound JR-FL gp120 (B). The β-14 (cyan), β-18 (pink) and β-24 strands (green) are highlighted. The CD4 binding loop is highlighted in purple. Asn362 (Thr378 in SIV) is labelled (cyan). Elements of the bridging sheet are highlighted in red. Potential hydrogen bond donors for N362 within the β-14, β-18 and β-24 strands are shown in (C) and are colored as in (A) and (B). CD4 residues contacting the CD4bs of gp120 are colored in yellow, and the molecular surface of Phe43 of CD4 is shown to illustrate the "Phe43 pocket" of the gp120 binding site of CD4. N362 is labelled and highlighted in red. Putative hydrogen bond partners are labelled in grey. Hydrogen bonds are depicted as dotted green lines. For simplicity, only the N362 hydrogen bond with R465 is shown.

Previous studies of brain-derived Envs identified the N283 variant in the C2 region of gp120 within one of the CD4 contact sites, which increases Env-CD4 affinity and enhances M-tropism [43]. Since blood-derived A-R5 viruses have enhanced M-tropism compared to PA-R5 viruses [34,36], and N362 is located immediately N-terminal to another CD4 contact site in the C3 gp120 region [Fig. 5 and [50]], we hypothesized that N362 may potentially affect Env structure and CD4 binding. N362, a potential site for N-linked glycosylation, was modelled on the unliganded crystal structure of SIV gp120 and CD4-liganded crystal structure of HIV-1 JRFL gp120. The CD4bs in the unliganded gp120 is located in the outer domain and consists of a disordered loop flanked by the β-14 and β-16 strands. N362 is positioned just distal to the β-14 strand in the disordered loop region of the CD4 binding motif (Fig. 7A). Upon binding to CD4, conformational changes lead to interactions between the β-14, β-18 and β-24 strands to form an antiparallel sheet (Fig. 7B), which is one of two major conformational changes that occur in gp120 upon CD4 binding [66]. Analysis of interatomic contacts within the antiparallel sheet region showed that N362 has the potential to form hydrogen bonds with one or more residues from within the β-14 strand and/or neighbouring strands of the β-sheet, including V360, F361, H363, F468, and R469 (Fig. 7C). Modeling glycosylated residues on the CD4-bound gp120 demonstrated N362 is in close proximity to N392, another potentially glycosylated residue in the β-18 strand (data not shown). Thus, N362 may be important in formation of the CD4-bound structure of gp120, and may contribute to the stability of the CD4-bound conformation of gp120 by forming intramolecular hydrogen bonds with residues from neighbouring strands and/or interaction with other glycosylated residues.

### A-R5 Envs with N362 have faster entry kinetics than PA-R5 Envs lacking N362

**Figure 8:**
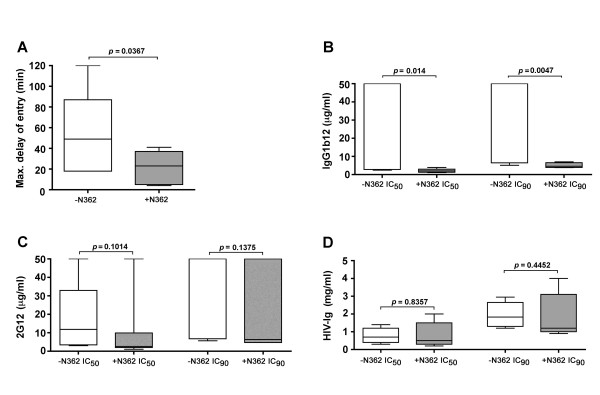
**The relationship between N362, HIV-1 entry kinetics, and sensitivity to inhibition by neutralizing antibodies**. Luciferase reporter viruses pseudotyped with a subset of PA-R5 Envs lacking N362 (NB24-C1, NB24-C2, NB24-C3, NB24-C4, NB25-C2, and NB25-C4) or with a subset of A-R5 Envs containing N362 (NB6-C2, NB6-C3, NB6-C4, NB7-C2, NB7-C4, NB8-C2 and NB8-C4) were produced and quantified as described in the Methods. The kinetics of HIV-1 entry by Env-pseudotyped luciferase reporter viruses was determined by time-of-addition studies with T-20, as described in the Methods (A). The results are expressed in minutes as the maximum delay time after addition of virus to JC53 target cells when addition of 50 μg per ml of T-20 can still completely inhibit HIV-1 entry (Max. delay of entry), which was determined by least squares regression analysis of time-of-addition curves. The sensitivity of Env-pseudotyped luciferase reporter viruses to neutralization by Env monoclonal antibodies IgG1b12 (B) or 2G12 (C), or by the polyclonal antibody HIV-Ig (D) was determined by calculation of IC_50 _and IC_90 _values by least squares analysis of neutralization curves, as described in the Methods. For sensitivity to neutralization by IgG1b12, four PA-R5 Envs (NB24-C1, NB24-C2, NB24-C3, NB24-C4) had IC_50 _and IC_90 _values > 50 μg per ml, indicating resistance. These Envs were assigned values of 50 μg per ml for the purpose of constructing panel (B). Box plots were constructed from mean values of duplicate experiments with each Env-pseudotyped luciferase reporter virus using Prism version 4.0c (GraphPad Software). Boxes represent upper and lower quartiles and median scores, and whiskers represent minimum and maximum values. The data shown are representative of 2 independent experiments. P values were calculated using a nonparametric Mann-Whitney *U *test, and values < 0.05 were considered statistically significant.

The structural models suggest N362 may influence CD4 binding and thus, the efficiency of HIV-1 entry. To better understand how N362 may enhance the entry of A-R5 Envs, we produced single-round luciferase reporter viruses pseudotyped with a subset of A-R5 Envs containing N362 (NB6-C2, NB6-C3, NB6-C4, NB7-C2, NB7-C4, NB8-C2 and NB8-C4) or with a subset of PA-R5 Envs lacking N362 (NB24-C1, NB24-C2, NB24-C3, NB24-C4, NB25-C2, and NB25-C4). Using time-of-addition studies with T-20, we compared the kinetics of HIV-1 entry between reporter viruses pseudotyped with A-R5 Envs containing N362 and PA-R5 Envs lacking N362 (Fig. 8A). In this assay, the maximal delay time after addition of virus to cells when addition of T-20 can still completely inhibit HIV-1 entry was measured; shorter delay times indicate faster entry kinetics, and longer delay times indicate slower entry kinetics. Viruses pseudotyped with Envs containing N362 had significantly shorter delay times than those pseudotyped with Envs lacking N362. Thus, the presence of N362 in the A-R5 Envs studied is associated with faster entry kinetics.

### A-R5 Envs with N362 have greater CD4bs exposure than PA-R5 Envs lacking N362

The prediction from structural models that N362 may contribute to stabilizing the CD4-liganded gp120 structure, and the observed faster entry kinetics by Envs containing N362 suggests that N362 may increase the exposure of the CD4bs in gp120. To determine the relationship between the presence of N362 in A-R5 Envs and CD4bs exposure in gp120, we compared the sensitivity of reporter viruses pseudotyped with A-R5 Envs containing N362 or PA-R5 Envs lacking N362 to neutralization by Env mAbs or HIV-Ig. Viruses pseudotyped with Envs containing N362 were more sensitive to neutralization by the Env mAb IgG1b12 than viruses pseudotyped with Envs lacking N362, as shown by significant differences in the IC_50 _and IC_90 _for IgG1b12 (Fig. 8B). In fact, 7/7 of the A-R5 Envs tested were highly sensitive to neutralization by IgG1b12 whereas in comparison, only 2/6 PA-R5 Envs were neutralized by IgG1b12 and 4/6 PA-R5 Envs were completely resistant to neutralization by IgG1b12 (IC_50 _and IC_90 _> 50 μg per ml). However, there were no differences in sensitivity to neutralization by the Env mAb 2G12 (Fig. 8C) or polyclonal HIV-Ig (Fig. 8D) between viruses pseudotyped with Envs either containing or lacking N362. Since the binding site for IgG1b12 overlaps the CD4bs in gp120 [67], these results suggest that the A-R5 Envs containing N362 have increased CD4bs exposure compared to the PA-R5 Envs lacking N362. This conclusion is supported by results of FACS-based antibody binding studies, which showed that A-R5 Envs containing N362 bound to non-saturating concentrations of IgG1b12 more efficiently than PA-R5 Envs lacking N362 when expressed to equivalent levels on the surface of 293T cells (data not shown).

### N362 contributes to enhanced fusogenicity of A-R5 Envs in a strain-dependent manner

**Figure 9:**
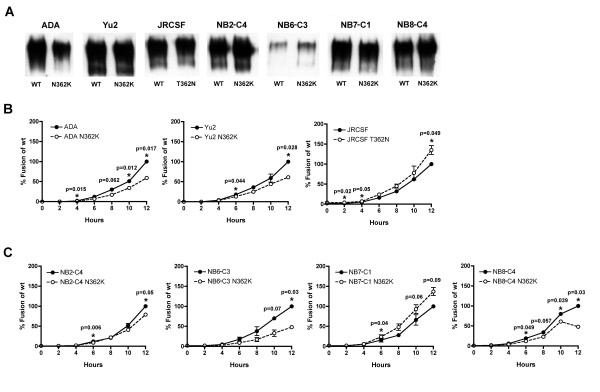
**N362 exerts a strain-dependent enhancement of fusogenicity by R5 Envs**. Western blot analysis of 293T cells transfected with wild type and Env mutants (A). Fusion assays were performed using 293T effector cells expressing equivalent levels of wild type or Env mutants generated from control R5 ADA, YU2 and JRCSF Envs (B) or from A-R5 NB2-C4, NB6-C3, NB7-C1 and NB8-C4 Envs (C), and Cf2-Luc target cells expressing CD4 and CCR5 as described in the Methods. Cells were harvested at 2, 4, 6, 8, 10 and 12 h post-mixing and assayed for luciferase activity. Results are expressed as the percentage of maximal fusion levels attained by the wild type Env clone. Means values of duplicate infections are shown. Error bars represent standard deviations. The results are representative of 2 independent experiments. P values were calculated with a paired t-test, and values < 0.05 were considered significant*. P values approaching significance are also indicated.

To determine whether N362 contributes to enhanced fusogenicity of R5 Envs, a panel of Env mutants was generated that either introduced or removed Asn at position 362 in gp120. Western blot analysis of transfected 293T cells demonstrated equivalent levels of Env expression between wild type Envs and Env mutants (Fig. 9A). The effect of N362 on fusogenicity was first determined in R5 control Envs (Fig. 9B). Wild type ADA and YU2 Envs have N362 and are highly fusogenic, whereas wild type JR-CSF Env lacks N362 and is, by comparison poorly fusogenic (data not shown). Replacement of Asn with Lys at position 362 in ADA and YU2 Envs resulted in significant reductions in fusogenicity. Conversely, introducing Asn at position 362 in JR-CSF Env resulted in a significant increase in fusogenicity. Thus, N362 contributes to enhanced fusogenicity of ADA and YU2 Envs and increases fusogenicity of JR-CSF Env.

To better understand the contribution of N362 to enhanced fusogenicity of A-R5 Envs, Asn at position 362 in gp120 of the highly fusogenic NB2-C4, NB6-C3, NB7-C1 and NB8-C4 Envs was replaced with Lys. The removal of Asn at position 362 resulted in significant reductions in fusogenicity of NB2-C4, NB6-C3 and NB8-C4 Envs, but resulted in an apparent increase in fusogenicity by NB7-C1 Env (Fig. 9C). However, increased fusogenicity by the NB7-C1 Env mutant was marginal and significant only at one early timepoint (6 h). Thus, whether substituting Asn for Lys at position 362 modulated fusogenicity of NB7-C1 Env is presently unclear. Together, results of the mutagenesis studies indicate N362 contributes to fusogenicity of the majority of the R5 Envs studied. However, the effect of N362 on fusogenicity of A-R5 Envs appears to be strain-dependent, suggesting the presence of additional factors contributing to fusogenicity of R5 Envs.

## Discussion

In this study we generated and characterized full-length, functional R5 Env clones derived from well characterized R5 HIV-1 viruses isolated from patients with asymptomatic infection or AIDS. In this panel of Envs, enhanced fusogenicity was a phenotype that distinguished A-R5 from PA-R5 Envs. We showed N362 near the CD4bs in the C3 region of gp120 occurs at higher frequency in A-R5 Envs than PA-R5 Envs and is associated with enhanced fusogenicity, decreased sensitivity to the inhibitory effects of the fusion inhibitor T-20, and increased HIV-1 entry kinetics. Mutagenesis studies showed N362 enhances fusogenicity of A-R5 Envs in a strain-dependent manner. Structural models indicate N362 is located adjacent to the CD4 binding loop of gp120, and together with conformational mapping studies with Env Abs suggest N362 may contribute to enhancement of fusogenicity by promoting greater exposure of the CD4bs and/or stabilizing the CD4-bound Env structure. Together, our results provide evidence that A-R5 Envs have genetic and structural alterations that augment Env-mediated fusion and entry. Enhanced fusogenicity may contribute to pathogenicity of late emerging R5 HIV-1 strains in subjects who persistently maintain R5 HIV-1 variants at late stages of HIV-1 infection.

Increased Env-mediated fusogenicity was found to be a prominent phenotype of the A-R5 Envs studied. Consistent with previous studies that showed membrane fusing capacity to be essential for Env-mediated cytopathicity *in vitro *[17], the results of our studies suggest increased fusogenicity by A-R5 Envs may reflect an increased ability of A-R5 Envs to cause cytopathic effects. This idea is supported by macaques studies, where passage of chimeric SHIV strains led to enhancement of pathogenicity associated with adaptive changes in Env [18-23]. These mutations arose in the gp120 C2, C3, V3, V4, and gp41 Env regions, resulting in increased Env-mediated fusogenicity that was thought to occur via increased Env-receptor binding. Thus, increased fusogenicity contributes to viral pathogenicity in the macaque model. In addition, increased cytopathicity by an A-R5 Env in SCID-hu mice was reported recently, and thought to occur via increased CCR5 usage [68]. The cytopathic effects of Env-mediated fusogenicity are also evident in humans as MNGC, which are present in autopsy brain tissues of subjects with HIVE [69] and formed by Env-mediated fusion between infected and uninfected macrophage-lineage cells [27]. Multinucleated giant cells in brain are caused predominantly by R5 HIV-1 Envs [44-46,70], which share several features with blood-derived A-R5 Envs such as enhanced fusogenicity [45,46,71], increased sensitivity to neutralization by IgG1b12 [45], and Env structures that enable efficient Env-CD4 interactions [43]. Thus, increased fusogenicity of blood-derived A-R5 Envs may enhance their cytopathic potential. This provides, at least in part, a plausible explanation contributing to CD4+ T-cell depletion that occurs in HIV-1-infected individuals who progress to AIDS whilst exclusively harbouring R5 HIV-1 variants.

Sequence analysis identified the presence of N362 near CD4bs residues in the C3 region of gp120 at higher frequency in A-R5 Envs than PA-R5 Envs. N362 was associated with enhanced fusogenicity and increased HIV-1 entry kinetics, suggesting that its presence contributes to the highly fusogenic, A-R5 Env phenotype. Molecular modeling the N362 residue on the unliganded SIV and CD4-liganded HIV-1 JRFL crystal structures places N362 immediately adjacent to the disordered loop region of the CD4bs in the unliganded gp120. In the CD4-liganded gp120, N362 has the potential to form hydrogen bonds with residues from neighbouring strands of the β-sheet. Alternatively, modeling glycosylated residues on the CD4-bound gp120 demonstrated N362 is proximal to N392, another potentially glycosylated residue in the β-18 strand (data not shown), suggesting N362 may influence the CD4-bound Env structure via interaction with other N-linked glycans. The latter possibility is supported by recent studies that showed the potentially glycosylated N386 residue in the V4 gp120 region influences CD4bs and IgG1b12 epitope exposure of certain brain-derived Envs [72]. These models suggest that N362 may contribute to stabilizing the CD4-bound state of gp120 by forming intramolecular hydrogen bonds with residues from neighbouring strands and/or interaction with other glycosylated residues.

In this context A-R5 Envs with N362 may have an enhanced ability to interact with CD4, which is supported by recent studies of the N283 Env variant that is present at high frequency in brain-derived R5 Envs [43,73], and was shown to enhance CD4 binding by forming an additional hydrogen bond with Gln 40 of CD4 [43]. Further supporting this hypothesis are results from recent studies showing increased CD4 affinity by engineered trimeric gp120 glycoproteins with enhanced IgG1b12 epitope exposure [74]. Studies of single-molecule bond force spectroscopy in living cells demonstrated that gp120-CD4 binding is short-lived and weak compared with gp120-CD4 complex binding to CCR5 [75], so enhanced Env-CD4 binding may increase the capacity of A-R5 Envs to use CD4 on target cells for HIV-1 entry. Alternatively, since CCR5 is more mobile in the cell membrane than CD4 [76], a more stable gp120-CD4 interaction by A-R5 Envs could potentially permit the Env-CD4 complex to more readily colocalize with CCR5, thus increasing the efficiency of CCR5 usage. Both of these possibilities are supported by our previous studies on the primary R5 HIV-1 isolates used to generate the A-R5 and PA-R5 Env clones, which showed that A-R5 isolates have reduced dependence on both CD4 and CCR5 levels for HIV-1 entry compared to PA-R5 isolates [36]. The possibility that A-R5 Envs may have enhanced CCR5 usage via increased Env-CD4 interactions is supported by recent studies that demonstrated enhanced cytopathicity of an A-R5 Env clone in SCID-hu mice that was thought to occur via increased CCR5 usage [68]. Additional protein binding studies are required to determine whether A-R5 Envs with N362 have increased CD4 binding. However, our results indicating that A-R5 Envs with N362 have greater binding to the Env mAb IgG1b12, which has been mapped to an epitope overlapping the CD4bs [67], and are more sensitive to neutralization by IgG1b12 than PA-R5 Envs lacking N362 support this contention.

Like the effect of the N283 variant in brain-derived R5 Envs on CD4 binding and M-tropism [43], the effect of N362 on augmenting Env-mediated fusogenicity of blood-derived R5 Envs appears to be context dependent, since it did not enhance fusogenicity of all A-R5 Envs (Fig. 9C). Furthermore, a subset of published A-R5 Envs (26%) lack N362, and a considerable fraction of published PA-R5 Envs (49%) contain N362 [34,38,58-65]. There were no other signature changes that segregated PA-R5 and A-R5 Envs, suggesting that cooperative changes that might be required for N362 to mediate enhanced fusogenicity are likely to be strain specific. Cooperative changes may include alterations in hydrogen bond partners of N362 which may affect the ability of N362 to stabilize the CD4-bound Env structure. The relationship between fusogenicity and CD4bs exposure attributable to N362 also appears to be context dependent, since comparisons of fusogenicity and sensitivity to neutralization by IgG1b12 between primary Envs and respective N362 Env mutants (Fig. 9C) showed a direct relationship between alterations in fusogenicity by N362 and sensitivity to IgG1b12 for NB6-C3 and NB7-C1 Envs, but not for NB2-C4 and NB8-C4 Envs (data not shown). Thus, the ability of N362 to increase fusogenicity of NB2-C4 and NB8-C4 Envs (Fig. 9C) may depend on other factors to increase exposure of the IgG1b12 epitope. Sequence analysis of the sequentially obtained Envs from subject IK1 demonstrated N362 in both the PA-R5 and A-R5 Envs (data not shown). However, the PA-R5 virus from IK1 differs from the PA-R5 viruses from the cross sectional panel in that it was isolated just prior to the onset of CD4+ T-cell loss and progression toward AIDS [13], whereas the cross sectional panel of PA-R5 viruses were isolated from subjects at earlier stages of asymptomatic infection [34]. Therefore, although the A-R5 viruses from both panels were isolated from patients with AIDS, there is considerable heterogeneity among the PA-R5 viruses across the panels with respect to the stage of asymptomatic HIV-1 infection from which they were isolated. It is possible that N362 may occur with different frequencies in PA-R5 Envs isolated at different stages of asymptomatic infection. Of note, even though the A-R5 Envs from IK1 were more fusogenic than PA-R5 Envs from this subject, the fusogenicity of the PA-R5 Envs from IK1 was comparable to that of the A-R5 Envs from the cross sectional cohort. Thus, although N362 is associated with enhanced fusogenicity of A-R5 Envs, additional Env changes present in A-R5 Envs of IK1 may increase fusogenicity further.

Whether R5 HIV-1 strains may acquire N362 through adaptive changes and contribute to the pathogenesis of R5 HIV-1 infection *in vivo *is presently unclear. However, in support of this hypothesis, longitudinal analysis of R5 Env sequences in two individuals infected from the same source showed maintenance of N362 in a rapid progressor whereas a nonprogressing subject continued to maintain a mixture of N362, K362, S362, T362 and F362 amino acid variants [64]. Thus, N362 may be selected *in vivo *and potentially contribute to progressive R5 HIV-1 infection in a host-dependent manner. It is also presently unclear whether phenotypic differences in R5 Envs attributable to N362 are likely to be relevant *in vivo*, since in some assays only relatively small differences were observed. More detailed longitudinal studies on virus derived from plasma of subjects with progressive R5 HIV-1 infection are necessary to determine the temporal nature of N362 acquisition and it's effect on HIV-1 pathogenicity *in vivo*.

In conclusion, enhanced fusogenicity is a phenotype of A-R5 Envs which is associated with N362 in gp120, and linked to increased entry kinetics and increased exposure of the CD4bs in gp120. N362 contributes to enhanced fusogenicity of A-R5 Envs in a strain-dependent manner. These results lead to a better understanding of the mechanisms contributing to CD4+ T-cell depletion in subjects who progress to AIDS whilst exclusively harbouring R5 HIV-1 variants.

## Methods

### Virus isolates

In this study, we utilized two independent panels of well characterized primary R5 HIV-1 isolates. The first was a cross sectional panel of R5 HIV-1 viruses isolated from immunocompetent subjects with asymptomatic HIV-1 infection (n = 4 isolates) or from patients with AIDS (n = 4 isolates) [34,36]. The second comprised R5 HIV-1 viruses isolated sequentially from one subject (IK1) from chronic HIV-1 infection to AIDS (n = 2 isolates) [13,42]. For the purpose of this study, the viruses isolated from patients with AIDS are referred to as AIDS R5 (A-R5) viruses, and those isolated from the earlier times are referred to as pre-AIDS R5 (PA-R5) viruses.

A detailed characterization of the cross sectional panel of R5 HIV-1 viruses including analysis of quasispecies diversity, coreceptor usage, replication kinetics, and clinical characteristics of the subjects from whom they were isolated, has been described previously [34,36]. Briefly, the PA-R5 viruses NB23, NB24, NB25 were isolated from peripheral blood mononuclear cells (PBMC) of individuals with CDC category II disease (asymptomatic infection) with CD4 counts of >500 cells/μl. PA-R5 virus NB27 was isolated from PBMC of an individual with CDC category I disease (acute seroconversion) with CD4 count of >750 cells/μl. A-R5 viruses NB2, NB6, NB7 and NB8 were isolated from PBMC of individuals with CDC category IV disease (AIDS) and CD4 counts of <50 cells/μl.

The longitudinal R5 HIV-1 viruses isolated from sequential PBMC samples from subject IK1 are designated IK1-PA and IK1-A. These viruses were referred to previously as 435–531 and 435–3415, respectively [13]. A detailed characterization of these HIV-1 isolates including analysis of quasispecies diversity, coreceptor usage, replication kinetics, and clinical characteristics of the subjects from whom they were isolated, has been described previously [13,42].

### Cells

PBMC were purified from blood of healthy HIV-1-negative donors, stimulated with 5 μg of phytohemagglutinin (PHA) (Sigma, St. Louis, MO) per ml for 3 days, and cultured in RPMI 1640 medium supplemented with 10% (vol/vol) fetal calf serum (FCS), 100 μg of penicillin and streptomycin per ml, and 20 U of interleukin-2 (IL-2) (Roche, Basel, Switzerland) per ml. Cf2-Luc cells [25] are derived from the Cf2th canine thymocyte cell line [77], and stably express the luciferase gene under the control of the HIV-1 long terminal repeat and were cultured in Dulbecco modified Eagle medium (DMEM) supplemented with 10% (vol/vol) FCS, 100 μg of penicillin and streptomycin per ml, and 0.7 mg of G418 per ml. Cf2-CD4 cells [78] were cultured in DMEM supplemented with 10% (vol/vol) FCS, 100 μg of penicillin and streptomycin per ml, and 0.5 mg of G418 per ml. Cf2-CD4/CCR5 cells [79] were cultured in DMEM supplemented with 10% (vol/vol) FCS, 100 μg of penicillin and streptomycin per ml, 0.5 mg of G418 per ml, and 0.1 mg of hygromycin per ml. Cf2-CD4/CXCR4 cells were constructed by transduction of the Cf2-CD4 cell line [78] with pBABE-puro vectors expressing CXCR4 [80,81] followed by selection and expansion in DMEM supplemented with 10% (vol/vol) FCS, 100 μg of penicillin and streptomycin per ml, 0.5 mg of G418 per ml, and 1 μg of puromycin per ml. Cf2-CD4/CCR5/CXCR4 cells [56] were cultured in DMEM supplemented with 10% (vol/vol) FCS, 100 μg of penicillin and streptomycin per ml, 0.5 mg of G418 per ml, 0.1 mg hygromycin per ml, and 1 μg puromycin per ml. JC53 cells are derived from the HeLa cell line and stably express high levels of CD4, CXCR4 and CCR5 on the cell surface [82], and were cultured in DMEM supplemented with 10% (vol/vol) FCS, and 100 μg of penicillin and streptomycin per ml. 293T cells were cultured in DMEM supplemented with 10% (vol/vol) FCS, and 100 μg of penicillin and streptomycin per ml.

### PCR amplification, HIV-1 Env cloning, identification of functional Envs, and sequence analysis

Genomic DNA was extracted from PBMC infected with NB2, NB6, NB7, NB8, NB23, NB24, NB25 and NB27 primary isolates using a QIAmp genomic DNA purification kit (Qiagen) according to the manufacturers' protocol. Viral RNA was isolated from 1 ml of IK1-PA and IK1-A primary isolates using a QIAmp UltraSense viral RNA isolation kit (Qiagen), according to the manufacturers' instructions. cDNA was reversed transcribed from viral RNA using SuperscriptIII RT (Invitrogen) and random hexamers, according to the manufacturers' protocol. An approximately 2.1 kb fragment spanning the *Kpn*I to *Bam*HI restriction sites in HIV-1 *env *(corresponding to nucleotides 6348 to 8478 in HXB2) was amplified by PCR using nested primers and Expand high fidelity DNA polymerase (Roche diagnostics), as described previously [56,57]. The outer primers were env1A and env1M [83], and the inner primers were Env-*Kpn*I and Env-*Bam*HI [56,57]. PCR cycling consisted of an initial denaturation step at 94°C for 2 min followed by 9 cycles of 94°C for 15 s, 60°C for 30 s and 72°C for 2 min, then a further 20 cycles of 94°C for 15 s, 60°C for 30 s and 72°C for 2 min but with a 5 s increasing extension time for each cycle, followed by a final extension at 72°C for 7 min. The products of 3 independent PCR reactions were purified and pooled, then cloned into the pSVIII-HXB2 Env expression plasmid [83] by replacement of the 2.1 kb *Kpn*I to *Bam*HI HXB2 *env *fragment. Thus, the resulting Env clones contain the entire gp160 coding region of primary virus-derived *env *genes except for 36 amino acids at the N terminus and 105 amino acids at the C terminus, which are derived from HXB2. Three to 4 functional Env clones from each virus were identified by the ability to support entry when pseudotyped onto Env-deficient GFP reporter viruses and used in single round entry assays in JC53 or Cf2-CD4/CCR5/CXCR4 cells, and by Western blot analysis of gp120/gp160 in transfected 293T cells and fusion assays. The coreceptor usage of functional Env clones was verified by single round entry assays in Cf2-CD4/CCR5 and Cf2-CD4/CXCR4 cells infected with Env-pseudotyped GFP reporter viruses, as described previously [56,84]. Envs were sequenced by Big Dye terminator sequencing (Applied Biosystems) and analyzed using a model 3100 Genetic Analyzer (Applied Biosystems).

### Env mutagenesis

Mutagenesis to introduce or remove Asn at position 362 in gp120 was performed using the QuikChange II site-directed mutagenesis kit (Stratagene) according to the manufacturer's instructions. Mutagenesis primers were designed to span the CD4 binding loop region corresponding to nucleotides 7297 to 7325 of HXB2 for control R5 Env clones ADA, YU2, and JR-CSF and primary A-R5 Env clones NB2-C4, NB6-C3, NB7-C1 and NB8-C4. The primers used in the mutagenesis studies are shown in Additional file 1. Envs mutants were sequenced to confirm the presence of the mutated residue.

### Western blot analysis

For analysis of Env expression, 293T cells were co-transfected with 8 μg of pSVIII-Env plasmid and 2 μg pSVL-Tat plasmid using the calcium phosphate method. At 72 h after transfection, cells were rinsed twice in PBS and resuspended in 400 μl of ice cold lysis buffer (0.5% [vol/vol] NP-40; 0.5% [wt/vol] sodium deoxycholate; 50 mM NaCl; 25 mM Tris-HCl [pH 8.0]; 10 mM EDTA, 5 mM benzamidine HCl; and a cocktail of protease inhibitors) for 10 min, followed by centrifugation at 15,300 × *g *for 10 min at 4°C to remove cellular debris. Cell lysates were separated in 8.5% (wt/vol) sodium dodecyl sulfate-polyacrylamide gel electrophoresis (SDS-PAGE) gels and analyzed by Western blotting using rabbit anti-gp120 polyclonal antisera. Env proteins were visualized using horseradish peroxidase-conjugated anti-rabbit immunoglobulin G antibody and enhanced chemiluminescence (Promega).

### Fusion assays

Fusion assays were conducted as described previously [43,45,71,85] with minor modifications. Briefly, 293T effector cells co-transfected with 3.4 μg of Env-expressing plasmid and 0.6 μg pSVL-Tat plasmid using Lipofectamine 2000 (Invitrogen) were mixed with Cf2-Luc target cells that had been co-transfected with 2 μg of pcDNA3-CD4 and 6 μg of pcDNA3-CCR5, and incubated at 37°C in replicate wells of 96-well tissue culture plates containing 200 μl of culture medium. Cells from replicate wells were harvested at 2, 4, 6, 8, 10 and 12 h post-mixing and assayed for luciferase activity (Promega) according to the manufacturers' protocol. 293T cells transfected with pSVL-Tat alone were used as negative controls to determine the background level of luciferase activity. In experiments analysing the ability of T-20 to inhibit Env function in fusion assays, the 10 h time point was used for analysis. To control for cell surface Env expression levels in 293T effector cells, Env expression was measured by flow cytometry using pooled AIDS serum and a FITC-conjugated anti-human F(ab')2 Ig (Chemicon). To account for both the number of Env expressing cells and the fluorescence intensity, the relative fluorescence was calculated from fluorescence-activated cell sorter (FACS) profiles by multiplying the percentage Env-expressing cells by the mean channel fluorescence, as described previously [86].

### Production and quantitation of Env-pseudotyped, luciferase reporter viruses

Env-pseudotyped, luciferase reporter viruses were produced by transfection of 293T cells with pCMVΔP1ΔenvpA, pHIV-1Luc and pSVIII-Env plasmids using Lipofectamine 2000 (Invitrogen) at a ratio of 1:3:1, as described previously [56,79,87,88]. Supernatants were harvested 48 h later and filtered through 0.45 μm filters. Recombinant luciferase reporter viruses were ultracentrifuged through a 25% (vol/vol) sucrose cushion at 25,000 rpm for 2 h at 4°C using a Beckman Ultra high speed centrifuge and a SW28 rotor, resuspended in 2 ml culture medium, aliquotted and stored at -80°C. The TCID_50 _of virus stocks was determined by titration in JC53 cells.

### Analysis of HIV-1 entry kinetics

Time-of-addition experiments using the HIV-1 fusion inhibitor T-20 and Env-pseudotyped luciferase reporter viruses were conducted to measure the entry kinetics of Env. This method was based on that recently described by Olivieri et al., [68] with the following modifications. Two hundred TCID_50 _of Env-pseudotyped luciferase reporter virus (equating to an MOI of 0.02) was added to replicate wells of JC53 cells. Fifty micrograms of T-20 per ml was added to the replicate wells at 0, 10, 20, 30, 40, 50 60 and 120 min post-infection. This concentration of T-20 was empirically determined to be completely inhibitory for each of the Env-pseudotyped luciferase reporter viruses tested when added to virus before infection of JC53 cells (data not shown). After 2 h, the virus inoculum was removed and the cells were washed twice prior to addition of fresh culture medium containing 50 μg of T-20 per ml. Cells were harvested 48 h later and assayed for luciferase activity (Promega) according to the manufacturers' protocol. The maximum delay time after addition of virus to JC53 cells when addition of 50 μg per ml of T-20 can still completely inhibit HIV-1 entry was calculated (Max. delay of entry). For these analyses, inhibition of entry was defined as luciferase activity measurements <3-fold above background, as determined using reporter virus pseudotyped with a non-functional Env (ΔKS Env) [25]. Luciferase activity was plotted against time of T-20 addition using Prism version 4.0c (GraphPad Software, San Diego, CA.). Data were fitted with a nonlinear function, and Max. delay of entry was calculated by least squares regression analysis of time-of-addition curves. Envs with lower Max. delay of entry values were interpreted to have faster entry kinetics, and vice-versa.

### Neutralization assays

Human mAbs against HIV-1 gp120 (IgG1b12, 2G12) and the polyclonal antibody HIV-Ig have been described previously [89-93]. The ability of these antibodies to neutralize the infectivity of Env-pseudotyped luciferase reporter viruses was assayed using JC53 cells. Two hundred TCID_50 _of each Env-pseudotyped luciferase reporter virus (equating to an MOI of 0.02) was incubated with 10-fold increasing concentrations of each mAb (0.0005 to 50 μg/ml) or HIV-Ig (1 to 10,000 μg/ml) for 1 h at 37°C. The virus-Ab mixtures were then used to inoculate JC53 cells overnight at 37°C. Cells were rinsed twice with culture medium to remove residual virus inoculum and incubated a further 48 h at 37°C. Virus infectivity was then measured by assaying luciferase activity in cell lysates (Promega), according to the manufacturers' protocol. Negative controls included mock-infected cells that were incubated with culture medium instead of virus, and cells treated with luciferase reporter virus pseudotyped with the non-functional ΔKS Env. After subtracting background luciferase activity, the amount of luciferase activity in the presence of antibody was expressed as a percentage of the amount produced in control cultures containing no antibody. The percent inhibition was calculated by subtracting this number from 100. Data were fitted with a nonlinear function, and fifty percent inhibitory concentration (IC_50_) and IC_90 _values were calculated by least squares regression analysis of inhibition curves, as described previously [36,45,85].

### Structural modeling

Proteins structures of unliganded SIV gp120 (2BF1) [66,94] and the V3 loop-containing protein structure of JR-FL (2B4C) [95] were obtained from the RCSB Protein Data Bank. Structural modelling was performed using Swiss PDB Viewer. Hydrogen bond analysis was performed using CSU software [96].

### Nucleotide sequence accession numbers

The Env nucleotide sequences reported here have been assigned GenBank accession numbers EU308533 to EU308568.

## Competing interests

The author(s) declare that they have no competing interests.

## Authors' contributions

JS, MJC, LRG and SS carried out the Env cloning; JS and AE performed fusion assays; JS, AE and MR produced Env-pseudotyped reporter viruses and conducted entry assays; JS, RLD and DG performed structural analyses; NS and BW performed the Env sequencing; SLW, IK, E-MF and ALC contributed clinical data; DFJP supplied essential reagents and contributed intellectually, JS and PRG designed the study, interpreted the data and wrote the manuscript; all authors helped edit the manuscript and have read and approved the final version.

## Supplementary Material

Additional file 1 Primers used for Env mutagenesis. The sequences of the oligonucleotide primers used to synthesize Env mutants are shown. Click here for file
