# Supplementary figures and images for: Asn 362 in gp120 contributes to enhanced fusogenicity by CCR5-restricted HIV-1 envelope glycoprotein variants from patients with AIDS
Source: Retrovirology. 2007 Dec 12;4:89. doi: 10.1186/1742-4690-4-89 (PMC2225424; doi:10.1186/1742-4690-4-89)

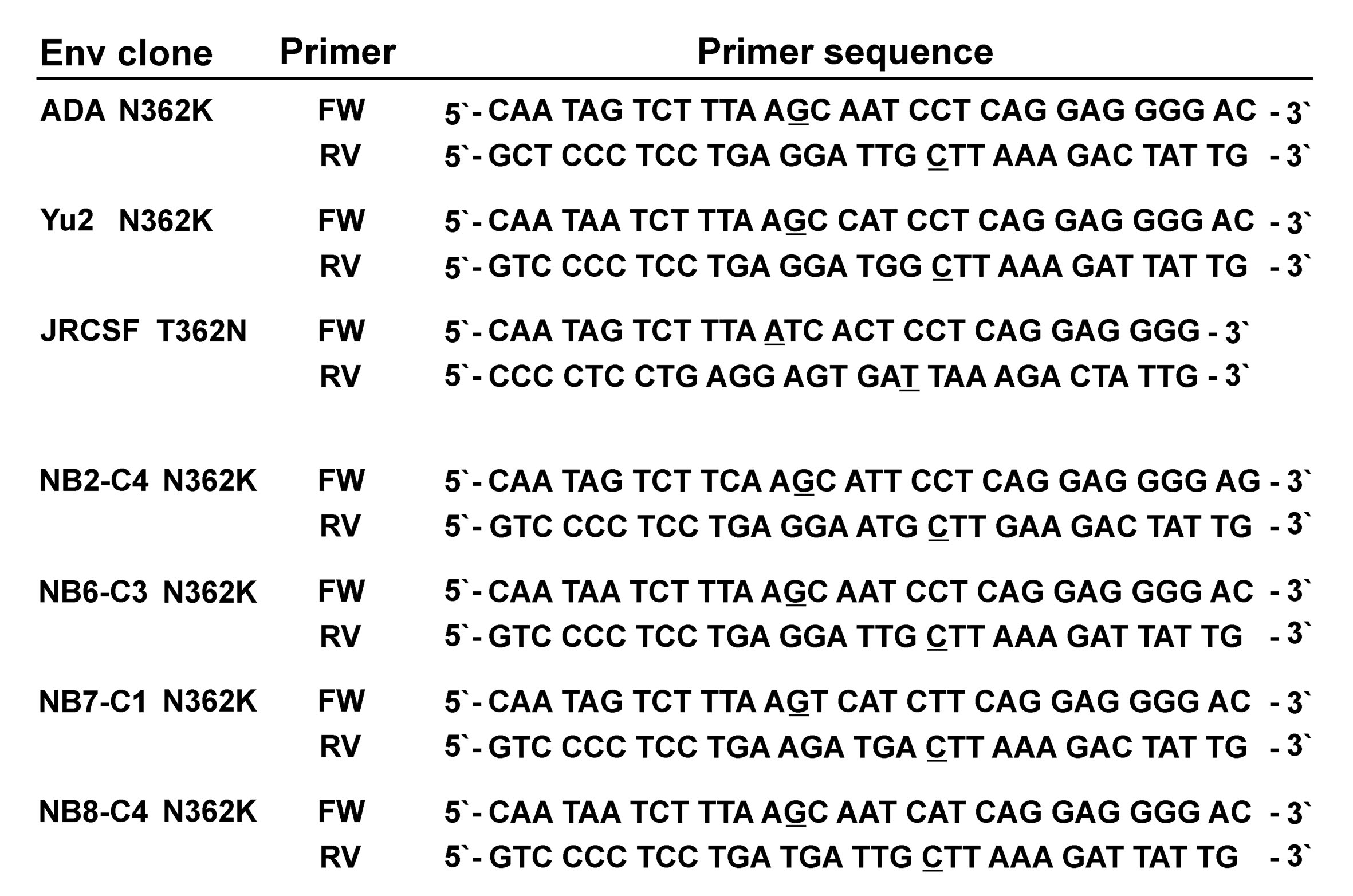

Supplement: Additional file 1 — Primers used for Env mutagenesis. The sequences of the oligonucleotide primers used to synthesize Env mutants are shown. [file 1742-4690-4-89-S1.jpeg]
